# Neuroligin-1 Loss Is Associated with Reduced Tenacity of Excitatory Synapses

**DOI:** 10.1371/journal.pone.0042314

**Published:** 2012-07-31

**Authors:** Adel Zeidan, Noam E. Ziv

**Affiliations:** Department of Physiology and Biophysics and Rappaport Institute, Technion Faculty of Medicine, and Network Biology Research Laboratories, Lorry Lokey Center for Life Sciences and Engineering, Haifa, Israel; Virginia Commonwealth University Medical Center, United States of America

## Abstract

Neuroligins (Nlgns) are postsynaptic, integral membrane cell adhesion molecules that play important roles in the formation, validation, and maturation of synapses in the mammalian central nervous system. Given their prominent roles in the life cycle of synapses, it might be expected that the loss of neuroligin family members would affect the stability of synaptic organization, and ultimately, affect the tenacity and persistence of individual synaptic junctions. Here we examined whether and to what extent the loss of Nlgn-1 affects the dynamics of several key synaptic molecules and the constancy of their contents at individual synapses over time. Fluorescently tagged versions of the postsynaptic scaffold molecule PSD-95, the AMPA-type glutamate receptor subunit GluA2 and the presynaptic vesicle molecule SV2A were expressed in primary cortical cultures from Nlgn-1 KO mice and wild-type (WT) littermates, and live imaging was used to follow the constancy of their contents at individual synapses over periods of 8–12 hours. We found that the loss of Nlgn-1 was associated with larger fluctuations in the synaptic contents of these molecules and a poorer preservation of their contents at individual synapses. Furthermore, rates of synaptic turnover were somewhat greater in neurons from Nlgn-1 knockout mice. Finally, the increased GluA2 redistribution rates observed in neurons from Nlgn-1 knockout mice were negated by suppressing spontaneous network activity. These findings suggest that the loss of Nlgn-1 is associated with some use-dependent destabilization of excitatory synapse organization, and indicate that in the absence of Nlgn-1, the tenacity of excitatory synapses might be somewhat impaired.

## Introduction

The formation of a synapse in the mammalian CNS is a complex, dynamic process that involves the coordinated differentiation of pre- and postsynaptic compartments (typically along axons and dendrites, respectively). These coordinated changes depend on the exchange of bidirectional signals, many of which are mediated by molecules that span the gap between the two compartments. Over the last decade, several classes of such molecules have been identified [1]–[4]. One prominent class is the Neuroligin (Nlgn) family (reviewed in [5]), which, in mammals, includes four to five genes [6]–[10]. Nlgn-1, Nlgn-2/4 and Nlgn-3 are postsynaptic, integral membrane cell adhesion molecules, mainly localized at excitatory, inhibitory, or both types of synapses, respectively [11]–[17]. The binding of postsynaptic neuroligins to presynaptic molecules belonging to the Neurexin (Nrxn) family has been shown to trigger the initial formation of synapses or play important roles in their subsequent maturation [5], [18], [19].

Perhaps the best studied member of the Nlgn family is Nlgn-1. Nlgn-1 was the first adhesion molecule ever shown to possess a specific capacity to induce presynaptic differentiation [20]. The extracellular domain of Nlgn-1 binds the extracellular domain of presynaptic Nrxn-1β, forming a trans-synaptic complex [6], [21], whereas its intracellular, cytoplasmic domain binds to postsynaptic density-95 (PSD-95; [22]), a key scaffolding protein of glutamatergic synapses that interacts with various glutamate receptors, signal transduction and cytoskeletal molecules (reviewed in [23], [24]). Nlgn-1, along with Nrxn-1β, has been suggested to play instrumental roles in recruiting and regulating the levels of N-methyl-D-aspartic acid (NMDA) receptors [25]–[27] and α-amino-3-hydroxy-5-methyl-4-isoxazolepropionic acid (AMPA) receptors [28]–[30]. Somewhat surprisingly, in spite of much evidence concerning Nlgn-1's importance for appropriate synapse formation, validation, and maturation [15], [20], [26], [28], [31]–[35], the knockout of Nlgn-1, alone or in combination with Nlgn-3, has only minor effects on synapse density, size, and numbers [13], [36], [37].

Much of what has been learned on the functional roles of Nlgn-1 was based on the manipulation of Nlgn-1 expression levels and the subsequent analysis of fixed tissues (electron microscopy, immunolabeling) or synaptic physiology. Given the multiple interactions of Nlgn-1 with postsynaptic molecules, on the one hand, and its transsynaptic interactions on the other, it is conceivable that the loss of Nlgn-1 might result in some destabilization of synaptic organization, and that these effects might go unnoticed in “single snapshot” analysis methods such as those mentioned above. For example, the loss of Nlgn-1 might adversely affect synaptic tenacity, that is, the capacity of individual synapses to maintain their particular characteristics over long durations, consequently accelerating the reversal of synaptic changes induced by physiological cues, or increasing the occurrence of spurious, spontaneous changes in synaptic properties. At present, however, little is known on relationships between Nlgn-1 and the stability or tenacity of excitatory synapses.

In the current study, we used primary cultures prepared from Nlgn-1 KO mice [13] and live imaging techniques to determine how the loss of Nlgn-1 affects the stability of postsynaptic densities, the constancy of presynaptic vesicle and postsynaptic glutamate receptor content as well as synaptic persistence over relatively long time scales. These experiments and their results are described next.

## Results

### Effects of Nlgn-1 loss on PSD-95 exchange rates and synaptic content constancy

**Figure 1:**
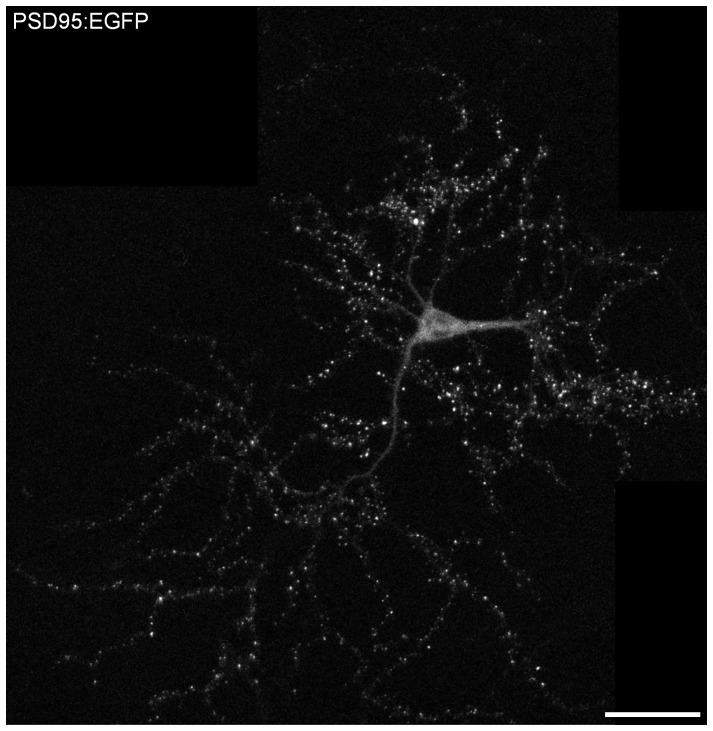
A mouse cortical neuron expressing PSD-95:EGFP. A composite image of a cortical neuron from a Nlgn-1 KO mouse expressing PSD-95 tagged with EGFP. Most bright puncta represent postsynaptic densities of dendritic spines. 17 days *in vitro*. Bar, 50 µm.

The intracellular C-terminal domain of Nlgn-1 has been shown to interact directly with the postsynaptic scaffolding molecule PSD-95 [22]. In addition, Nlgn-1 is believed to play a role in recruiting PSD-95 during synaptogenesis [26], [30]. If this interaction is central to the recruitment of PSD-95 to postsynaptic compartments, the loss of Nlgn-1 might therefore impact the dynamics of PSD-95 at postsynaptic sites. To examine if and to what degree the loss of Nlgn-1 affects PSD-95 dynamics, we prepared primary cultures of cortical neurons from homozygous WT and Nlgn-1 KO mice obtained from the same litters. A fusion protein of PSD-95 and EGFP (PSD-95:EGFP) was then expressed in these preparation by means of a third generation lentiviral vector [38], [39]. As shown in Fig. 1, PSD-95:EGFP assumed a punctate appearance, with puncta commonly located at the tips of dendritic spines. As previously demonstrated [39], [40], the vast majority of such puncta represent *bona fide* synapses. We then compared two major aspects of PSD-95:EGFP dynamics in preparations from WT and Nlgn-1 KO mice: PSD-95:EGFP exchange rates and postsynaptic remodeling.

**Figure 2:**
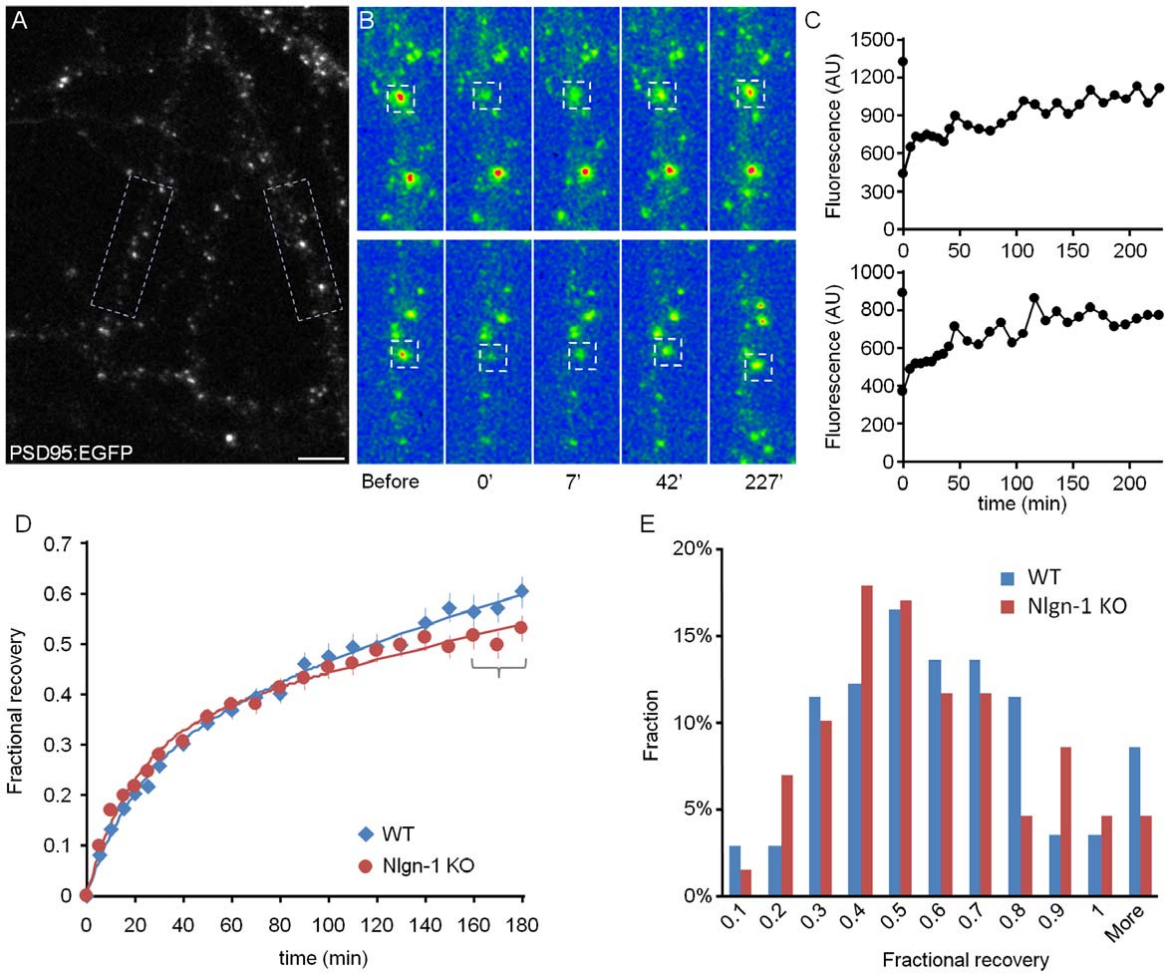
Exchange rates of PSD-95:EGFP at individual synapses. **A**) Dendrites of a neuron from a WT mouse expressing PSD-95:EGFP. Two PSD-95:EGFP puncta, one in each rectangular region, were selected for photobleaching. Bar, 10 µm. **B**) The two puncta (enclosed in squares) were selectively photobleached by high intensity laser light and fluorescence recovery at these sites was subsequently followed by time-lapse imaging. Fluorescence intensities shown in false color. **C**) Fluorescence recovery time course for the two photobleached puncta shown in A and B. **D**) Average FRAP curves for synapses from Nlgn-1 KO (13 neurons, 129 synapses) and WT littermates (13 neurons, 140 synapses; Mean ± SEM). Fits to sums of two exponentials (see Results) are shown as thick lines. **E**) Distribution of fractional recovery values for all photobleached PSD-95:EGFP puncta. The fractional recovery of each punctum was calculated as the average value of the 3 last points of its normalized recovery curve (as indicated by curly bracket in D).

To evaluate how the loss of Nlgn-1 affects PSD-95:EGFP exchange rates, we performed fluorescence recovery after photobleaching (FRAP) experiments. To that end, neurons from WT or Nlgn-1 KO mice, (maintained in culture for at least two weeks) were placed on a custom-built confocal laser scanning microscope, maintained at 36°C and provided with a sterile air mixture of 5% CO_2_ and 95% air. Following the collection of baseline images, fluorescence in PSD-95:EGFP was photobleached by high intensity laser light confined to small regions of interest centered on these puncta. Fluorescence recovery was then recorded for 3 hours, initially at 5 min intervals and later at 10 min intervals (Fig. 2A–C). Following the experiments, FRAP data was normalized, corrected for ongoing photobleaching ([41]; see methods) and the recovery data was fit to a weighted sum of two exponentials according to the following equation ([41]; see methods):


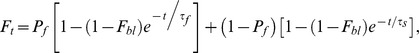


where *P*_f_ is the fractional size of the fast pool, *F*_bl_ is the normalized fluorescence immediately after the photobleaching procedure, and τ_f_ and τ_s_ are recovery time constants for “fast” and “slow” pools, respectively.

As shown in Fig. 2D fluorescence recovery was rather slow (Nlgn-1 KO: τ_f_ = 18 min, τ_s_ = 7.2 hr, P_f_ = 0.30; 13 neurons, 129 synapses; WT: τ_f_ = 20 min, τ_s_ = 4.7 hr, P_f_ = 0.24, 13 neurons, 140 synapses), and was not complete even after 3 hours. The extent of recovery was highly variable from one synapses to another (in agreement with [42]), with some synapses showing nearly full recovery after 3 hours while others showed very little recovery over the same period. To quantify the recovery at the end of this period, we averaged the last three points of the recovery curve of each synapse, plotted the distribution of these values (Fig. 2E), and subjected the distributions obtained in Nlgn-1 KO and WT neurons to a statistical test, which indicated that the differences were not statistically significant (p = 0.23, Kolmogorov-Smirnov test). These finding thus suggest that the loss of Nlgn-1 did not significantly impact the exchange rates of synaptic PSD-95:EGFP.

PSD-95 is a major postsynaptic scaffold protein of glutamatergic synapses and therefore, changes in PSD-95:EGFP fluorescence almost certainly reflect changes in postsynaptic density size. Therefore, assessing the stability of the PSD-95:EGFP fluorescence intensity at a particular synapse provides a readout of postsynaptic size stability of that particular synapse [39], [43]. More conservatively, the constancy of PSD-95:EGFP fluorescence intensity provides a measure of the constancy of PSD-95 content at a particular synapse.

**Figure 3:**
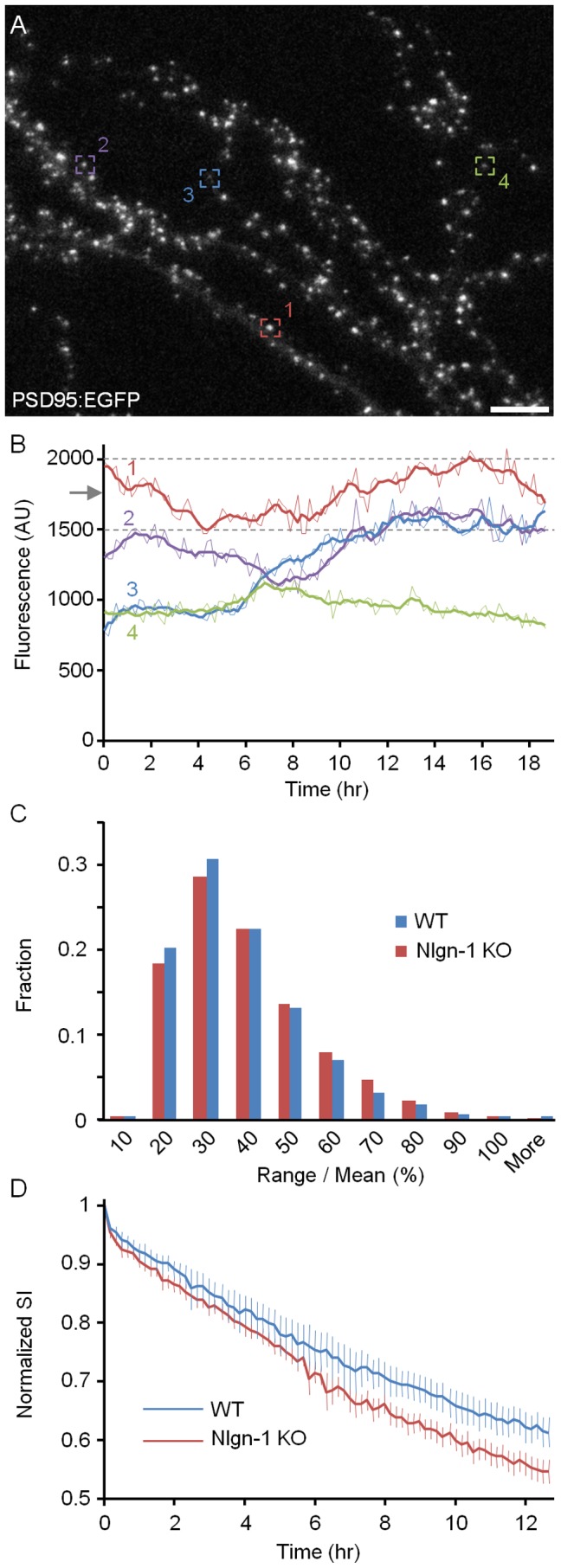
Constancy of PSD-95:EGFP contents at individual synapses. **A**) Dendrites of a neuron from a Nlgn-1 KO mouse expressing PSD-95:EGFP (23 days *in vitro*). Full time lapse sequence of this region is shown in Video S1. Bar, 10 µm. **B**) Changes over time in the fluorescence intensity of four puncta (enclosed in numbered and color coded rectangles) over >18 hours. Raw data is shown as thin lines whereas thick lines show the same data after smoothing with a 5 point low pass filter. A calculation of the range over mean measure is illustrated for punctum #1: the dashed lines show the minimal and maximal values of the smoothed data and the arrow indicates the mean fluorescence intensity over the imaging period. **C**) Distribution of range over mean values for all PSD-95:EGFP puncta measured in these experiments (Nlgn-1 KO: 46 neurons, 3465 puncta; WT: 44 neurons, 3367 puncta). **D**) SI decay rates measured in neurons from Nlgn-1 KO and WT mice (mean ± SEM).

To assess if and to what degree the loss of Nlgn-1 affects the constancy of PSD-95 content at individual synapses, neurons from WT or Nlgn-1 KO mice were mounted as described above, and imaged at 10 min intervals for at least 12 h. After the experiments, individual PSD-95:EGFP puncta were tracked in the time-lapse image series, and the fluorescence of each was measured at all time points (Fig. 3A,B; see also Video S1). Only puncta that could be indentified and tracked reliably were included in this analysis; Puncta that split or merged were rejected. In this manner, the fluorescence intensity profiles of numerous PSD-95:EGFP puncta were collected (Nlgn-1 KO: 46 neurons, 3465 puncta; WT: 44 neurons, 3367 puncta).

To quantify the extent of changes individual synapses undergo we calculated the normalized range of PSD-95:EGFP fluorescence change (which will be referred to here as *range over mean*) for each synapse [44] as follows:


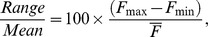


where *F_max_* is the maximal fluorescence intensity measured for a given synapse during the experiment, *F_min_* is its minimal fluorescence intensity, and 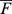 is its mean fluorescence value. To minimize the effects of measurement noise, the values were calculated after “smoothing” the data with a 5-point low-pass filter. The calculation of this measure is illustrated in Fig. 3B. The distributions of range over mean values in neurons from Nlgn-1 KO and WT mice are shown in Fig. 3C. As shown in this figure, range over mean values in Nlgn-1 KO neurons were slightly increased in comparison to those measured in WT neuron (34.64±16% versus 33±16%; mean ± standard deviation for Nlgn-1 KO and WT, respectively; Nlgn-1 KO: 46 neurons, 3465 puncta; WT: 44 neurons, 3367 puncta; p<10^−3^, Kolmogorov-Smirnov test).

Range over mean values provide a measure of fluorescence instability, but do not separate between instantaneous fluctuations and long-term trends. We therefore applied a second measure, which quantifies the degree to which PSD-95:EGFP puncta along the same dendritic tree change relative to each other over the entire duration of the experiments, and thus exposes trends on longer time scales. To that end, we calculated the decay rate of the Similarity Index (SI; [44], [45]) as follows:


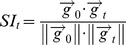


where 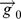 is a vector of fluorescence intensities of puncta belonging to a particular dendrite at time t = 0, and 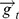 describes the fluorescence of the same puncta at time t. As SI is essentially the cosine of the angle between the two vectors, a value of 1.0 means no change in puncta brightness relative to each other (an angle of 0°) whereas increasingly smaller values indicate increasingly greater changes. Put differently, if the relative sizes of synapses along a dendritic tree can be viewed as a “synaptic configuration”, greater rates of SI decay signify poorer preservations of such synaptic configurations. Note that SI is not affected by multiplication of g_t_ with a scalar, (which only affects the vectors length, not its angle) and thus should not be affected by photobleaching (which can be approximated by multiplication of all fluorescence values by a constant <1.0). To reliably compare SI values from different cells, we normalized these to the minimum attainable SI value calculated for each neuron (see methods). As shown in Fig. 3D, the normalized SI measured in Nlgn-1 KO neurons decayed at somewhat faster rates as compared to that measured in WT neurons (to 0.56 and 0.62 by the end of the 12 hour period, Nlgn-1 KO and WT, respectively; p<0.05, Kolmogorov-Smirnov test; see Methods for details on statistical testing of differences between SI decay curves). This would seem to imply that the loss of Nlgn-1 is associated with a reduced constancy of PSD-95:EGFP contents at individual excitatory synapses and, probably with greater postsynaptic remodeling in these neurons.

**Figure 4:**
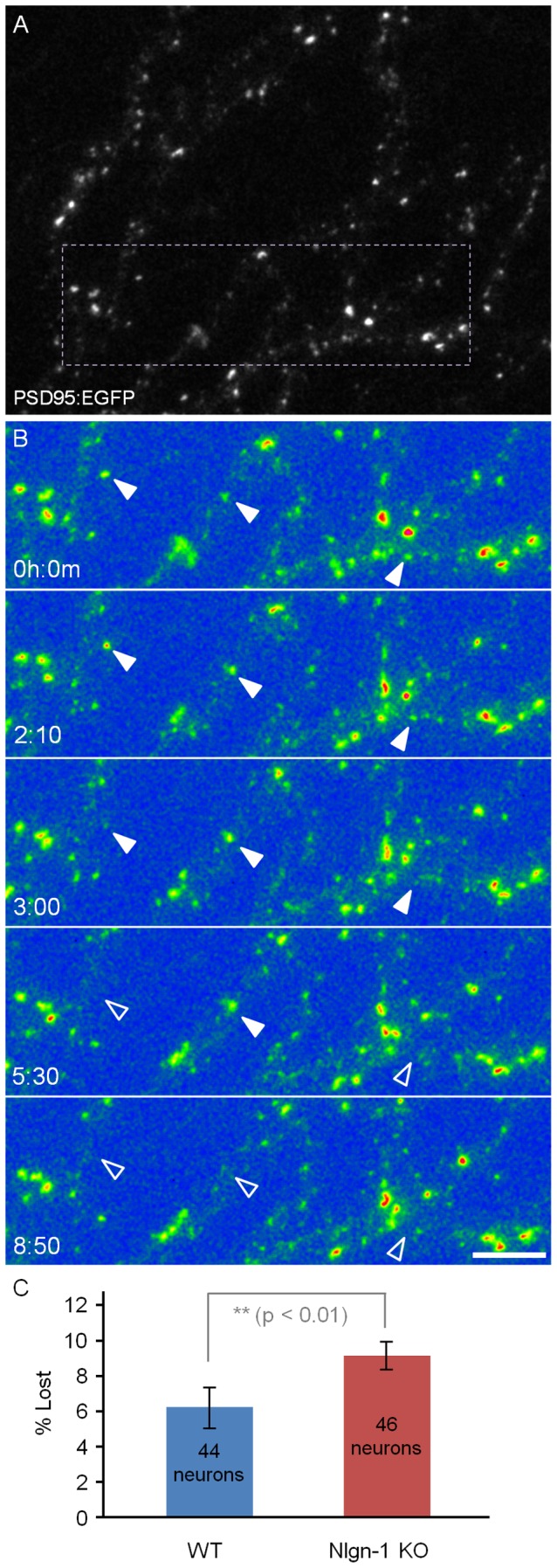
PSD-95:EGFP puncta loss rate. **A**) Dendrites of a neuron from a Nlgn-1 KO mouse expressing PSD-95:EGFP (21 days *in vitro*). **B**) Time lapse sequence of region enclosed in rectangle in A. Full arrowheads point to PSD-95:EGFP lost over time (empty arrowheads). Fluorescence intensities shown in false color. **C**) Fractional loss rates (% lost over 12 hour periods) in neurons from Nlgn-1 KO mice and WT littermates.

Finally, we quantified the fraction of PSD-95:EGFP puncta that could be reliably tracked but clearly disappeared during these time lapse sessions (as exemplified in Fig. 4). We found that the fraction of lost synapses was greater in neurons from Nlgn-1 KO mice as compared to WT neurons (9.1±5.5% versus 6.2±7.3%; mean ± standard deviation; 46 and 44 neurons, respectively; p<0.01, Kolmogorov-Smirnov test; Fig. 4C). In contrast, total synaptic numbers did not differ significantly between neurons from Nlgn-1 KO and WT mice (mean number of PSD-95:EGFP puncta/field of view 246±81 versus 260±114 respectively; mean ± standard deviation; p = 0.38, Kolmogorov-Smirnov test) These findings suggest that synaptic turnover rates are somewhat elevated in networks from Nlgn-1 KO mice, further indicating that the loss of Nlgn-1 adversely affects synaptic stability.

### Effects of Nlgn-1 loss on the constancy of presynaptic vesicle pools

**Figure 5:**
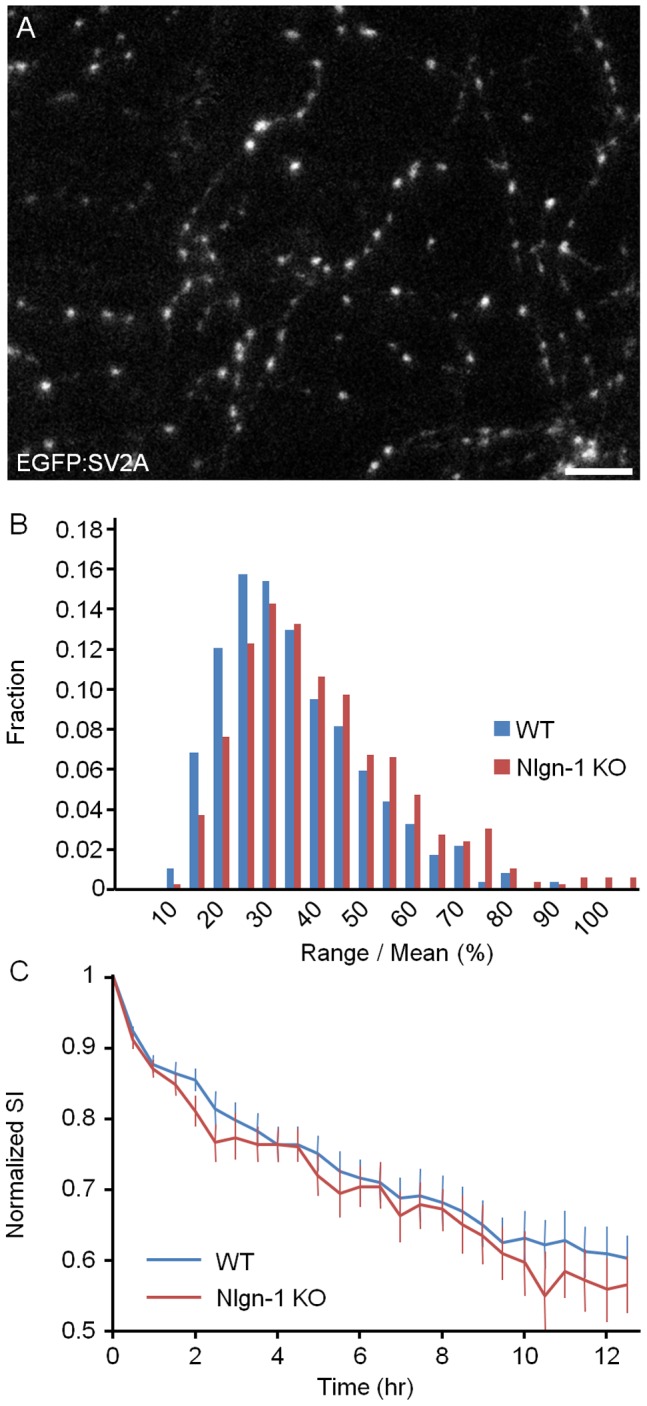
Constancy of synaptic vesicle contents at individual presynaptic sites. **A**) Axons of a neuron from a WT mouse expressing EGFP:SV2A (22 days *in vitro*). Individual fluorescent puncta represent presynaptic clusters of synaptic vesicles. Bar, 10 µm. **B**) Distribution of range over mean values for all EGFP:SV2A puncta followed in these experiments (Nlgn-1 KO: 19 neurons, 599 boutons; WT: 21 neurons, 856 boutons). **C**) SI decay rates measured in neurons from Nlgn-1 KO and WT mice (mean ± SEM).

Previous studies have shown that Nlgns, through their interactions with presynaptic β-Nrxns can induce presynaptic differentiation [e.g.20], [31], [32], [46], [47], modulate presynaptic vesicle release [48], regulate presynaptic maturation, and control the pool size of recycling synaptic vesicles [34], [49]. Given prior studies suggesting that synaptic vesicles are continuously interchanged among nearby synapses [50]–[52] and that these dynamics can significantly change the synaptic vesicle contents of individual synapses [44], [53], we wondered how the loss of Nlgn-1 might affect the rates of synaptic vesicle redistribution among neighboring synapses. To that end, we labeled synaptic vesicles using an EGFP-tagged variant of the synaptic vesicle protein SV2A (EGFP:SV2A) in a small set on neurons using a lentiviral vector ([44], [54]; Fig. 5A), and followed synaptic vesicle redistribution using time-lapse imaging for at least 12 hours at 10 min intervals as described above.

As shown in Fig. 5B, range over mean values measured over 12 hours were significantly greater in neurons from Nlgn-1 KO mice as compared to neurons from WT littermates (Nlgn-1 KO: 38±17%, 7 experiments, 19 neurons, 599 boutons; WT: 32±15%, 7 experiments, 21 neurons, 856 boutons; mean ± standard deviation; p<10^−6^, Kolmogorov-Smirnov test). On the other hand, no statistically significant difference was found for SI decay rates (p>0.8, Kolmogorov-Smirnov test).

In summary, the constancy of synaptic vesicle contents at individual synapses was slightly affected in neurons lacking Nlgn-1, indicating that the loss of Nlgn-1 is associated with some destabilization of presynaptic organization.

### Effects of Nlgn-1 loss on the constancy of synaptic AMPAR contents

AMPA – type glutamate receptors are indirectly associated with Nlgn-1 through PSD-95 and transmembrane AMPA receptor regulatory proteins (TARPs). Indeed, previous studies have demonstrated that Nlgn-1 play roles in AMPAR trafficking and recruitment [28]–[30]. Furthermore, newborn Nlgn-1 knockout mice exhibit lower levels of synaptic AMPARs and reduced AMPAR-dependent synaptic transmission [30]. Finally, Nlgn-1 KO exhibit reduced ratios of NMDA to AMPA receptor-mediated excitatory postsynaptic currents (EPSCs; [15], [36]) which seems to be age-dependent [55]. Given the extraordinary dynamics that AMPAR exhibit in terms of exo/endocytosis and, in particular, lateral diffusion between synaptic and extrasynaptic domains [56], [57], it is conceivable that the loss of Nlgn-1 might affect the constancy of AMPAR content at individual synapses.

**Figure 6:**
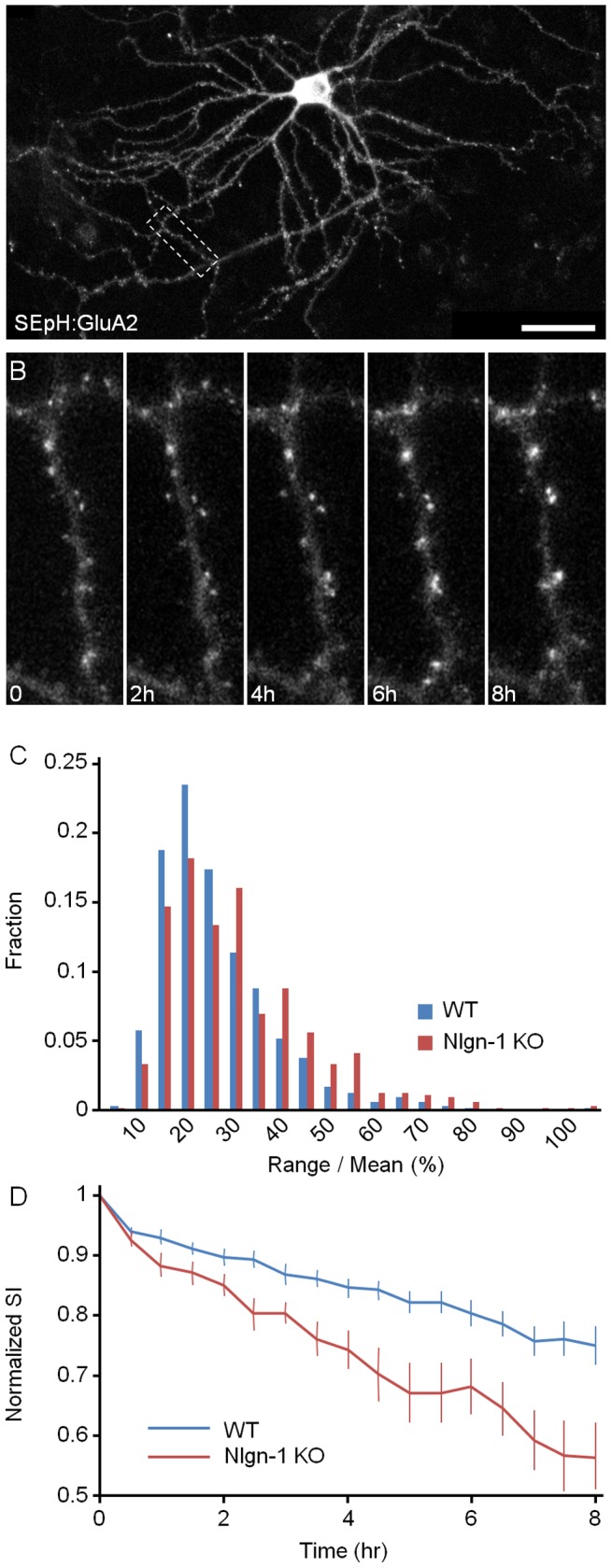
Constancy of AMPAR contents of individual postsynaptic sites. **A**) A composite image of a cortical neuron from a Nlgn-1 KO mouse expressing SEpH:GluA2. Most bright puncta are located on the postsynaptic membrane of dendritic spines. 19 days *in vitro*. Bar, 50 µm. **B**) Time lapse sequence of region enclosed in rectangle in A. Only a subset of images is shown here. **C**) Distribution of range over mean values for all SEpH:GluA2 puncta followed in these experiments (Nlgn-1 KO: 15 neurons, 563 synapses; WT: 14 neurons, 652 synapses). **D**) SI decay rates measured in neurons from Nlgn-1 KO and WT mice (mean ± SEM).

To quantify the stability of AMPAR content, we generated a fusion protein of the major AMPAR subunit GluA2 and superecliptic pHluorin (SEpH:GluA2; [58]–[60]), and expressed it in neurons from Nlgn-1 KO mice and from WT littermates using a lentiviral vector (Fig. 6A). AMPARs are composed of four subunits, with most receptors containing at least one GluA2 copy [61]–[63]. Moreover, the fluorescence of superecliptic pHluorin, when excited with blue light, is strongly pH dependent; thus receptors whose N-termini are in a low pH environment (for example lumenal domains of intracellular organelles such as lysosomes) will be practically invisible, whereas receptors on the cell surface (in which the N-termini face the extracellular space) will be fluorescent. Thus SEpH:GluA2 fluorescence can be used to quantify the AMPAR content of the postsynaptic membrane of individual synapses. Indeed, replacing the extracellular media with a physiological solution titrated to a pH of 5.5 leads to the immediate quenching of nearly all SEpH:GluA2 fluorescence, which recovers completely when the extracellular pH is restored to pH 7.4 (data not shown).

Time lapse imaging of neurons from Nlgn-1 KO and WT mice expressing SEpH:GluA2 was carried out for 8 h at 30 min intervals (Fig. 6B) and the data was subjected to a similar analysis as described for PSD-95:EGFP and EGFP:SV2A. Comparisons of range over mean value distributions revealed significantly greater values in neurons from Nlgn-1 KO mice as compared to those from WT littermates (Fig. 6C; Nlgn-1 KO: 28.3±16%, 5 experiments, 15 neurons, 563 synapses; WT: (23.6±13%, 5 experiments, 14 neurons, 652 synapses; mean ± standard deviation; p<10^−6^, Kolmogorov-Smirnov test). Similarly, the SI decayed significantly faster for neurons obtained from Nlgn-1 KO mice, with SI values reduced at the end of 8 hour periods to 0.57 and 0.75 in Nlgn-1 KO and WT neurons, respectively (Fig. 6D; p<0.04, Kolmogorov-Smirnov test).

**Figure 7:**
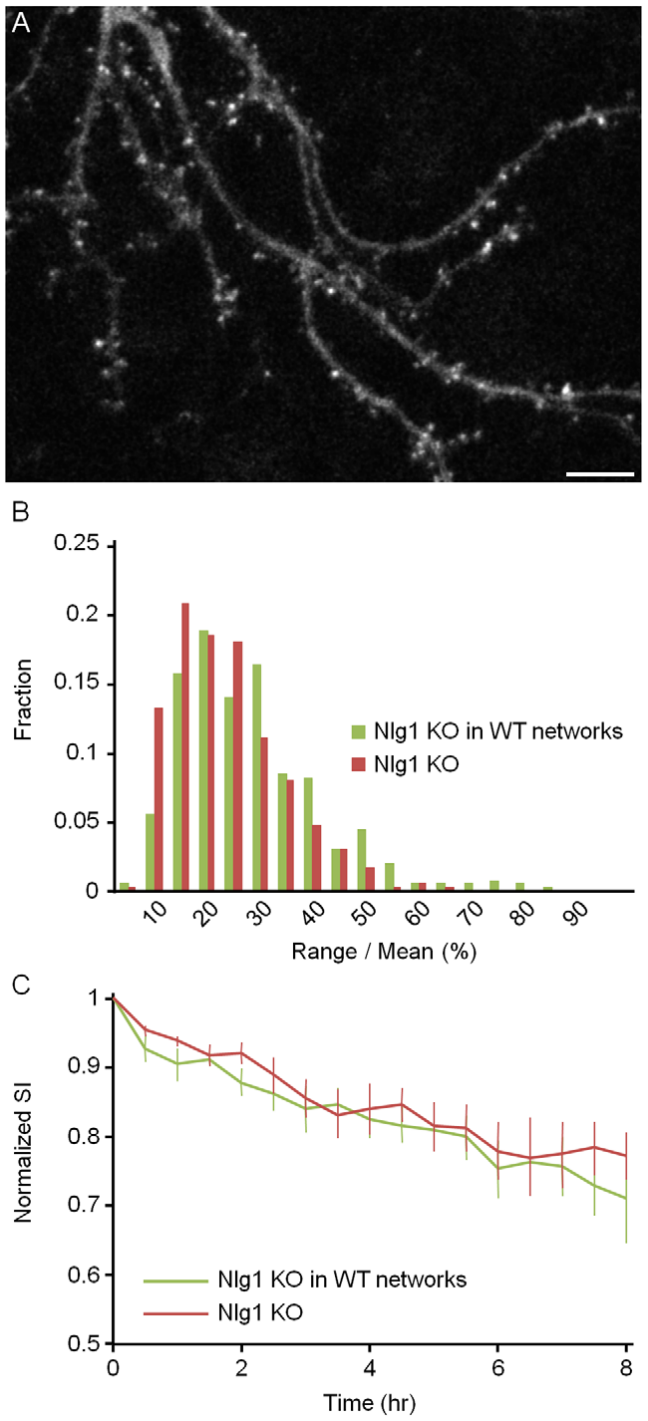
AMPAR redistribution dynamics of Nlgn-1 deficient neurons in a WT environment. **A**) A cortical neuron expressing SEpH:GluA2 from a Nlgn-1 KO mouse plated in a network in which the majority of cells were obtained from a WT littermate. 23 days *in vitro*. Bar, 10 µm. **B**) Distribution of range over mean values for all SEpH:GluA2 puncta followed in these experiments (Nlgn-1 KO neurons in WT neuronal networks: 6 neurons, 563 synapses; Nlgn-1 KO networks: 6 neurons, 652 synapses). **C**) SI decay rates (mean ± SEM).

Given the importance of Nlgn-Neurexin interactions we wondered if placing Nlgn-1 deficient neurons in an environment in which most other neurons have a normal complement of Nlgn-1 would exacerbate the instability of AMPAR contents, due to, for example, a poorer capacity to compete over axonal Neurexins. To that end we isolated neurons from Nlgn-1 KO mice, infected them with lentiviral particles encoding for SEpH:GluA2 and plated a minority of these neurons with a majority of neurons prepared in parallel from WT littermates (see Methods for details). After two weeks, paired experiments were performed as described above with mixed KO/WT networks and networks containing only Nlgn-1 KO neurons. Although range over mean values were somewhat higher in Nlgn-1 KO neurons growing among WT neurons (Fig. 7A; Nlgn-1 KO neurons in WT neuron networks: 26±14%, 3 experiments, 6 neurons, 563 synapses; Nlgn-1 KO networks: 21±10%, 3 experiments, 6 neurons, 652 synapses; mean ± standard deviation; p<10^−3^, Kolmogorov-Smirnov test), no significant differences were observed in SI decay rates in comparison to paired experiments performed in networks from Nlgn-1 KO mice (Fig. 7B; p>0.3, Kolmogorov-Smirnov test). It thus seems that AMPAR dynamics in these neurons are largely dictated by the genetic makeup of individual neurons.

**Figure 8:**
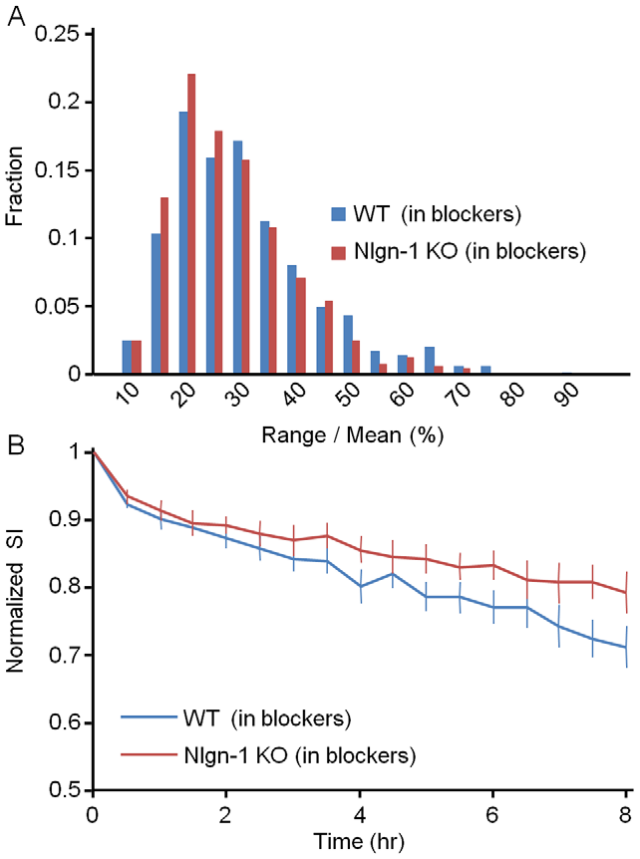
Activity dependence of AMPAR redistribution dynamics. **A**) Distribution of range over mean values for SEpH:GluA2 puncta in the presence of CNQX (10 µM), AP-5 (50 µM) and TTX (1 µM) (Nlgn-1 KO: 10 neurons, 481 synapses; WT: 10 neurons, 514 synapses). **B**) SI decay rates in the presence of the aforementioned pharmacological agents.

Interestingly, the effects of Nlgn-1 loss on AMPAR content constancy seemed to also depend on network activity: When experiments were performed in the presence of the glutamate receptor blockers CNQX (10 µM), AP-5 (50 µM), as well as TTX (1 µM), range over mean values (Fig. 8A) were actually *smaller* in networks prepared from Nlgn-1 KO mice as compared to networks prepared from WT littermates (Nlgn-1 KO: 25±11%, 5 experiments, 10 neurons, 481 synapses; WT: 28±13%, 5 experiments, 10 neurons, 514 synapses; mean ± standard deviation; p<0.03, Kolmogorov-Smirnov test). SI decay rates exhibited a similar trend (Fig. 8B) but it did not reach statistical significance (p>0.3, Kolmogorov-Smirnov test).

In summary, the loss of Nlgn-1 seems to be associated with an activity dependent impairment in the ability of individual synapses to maintain a constant content of AMPARs.

## Discussion

Here we describe experiments aimed at examining if and to what degree the loss of Nlgn-1 affects the dynamics of synaptic molecules and the constancy of synaptic contents of scaffolding molecules, synaptic vesicles and glutamate receptors. Using live imaging techniques and primary cultures from Nlgn-1 KO mice and WT littermates, we found that the synaptic contents of PSD-95:EGFP, EGFP:SV2A and SEpH:GluA2 exhibited greater changes over time in neurons lacking Nlgn-1 as compared to neurons from WT littermates, and a poorer preservation of PSD-95 and GluA2 contents of individual postsynaptic sites. Moreover, a somewhat greater degree of synaptic turnover was observed in Nlgn-1 KO neurons, manifested as reduced synaptic persistence in time-lapse experiments. Interestingly, these effects, at least where synaptic GluA2 contents were concerned, seemed to depend on spontaneous network activity. Taken together, these findings indicate that the loss of Nlgn-1 is associated with some use-dependent destabilization of synaptic organization, and that in the absence of Nlgn-1, the tenacity of excitatory synapses is somewhat impaired.

### Experimental considerations

The conclusions reached in this study were based on several experimental and analytical approaches, which warrant some discussion. First, all experiments were performed in primary cultures of dissociated neurons. Moreover, the experiments were carried out on networks (2–3 weeks in culture) that were still undergoing some degree of development. It thus remains possible that the absolute values reported here overestimate the dynamics of the same molecules in the adult brain. It is nevertheless interesting to note that PSD-95:EGFP exchange kinetics reported here are compatible or even slower than those measured *in vivo* [42]. Second, we noted that *absolute* range over mean values or SI decay rates tended to change over time even for the same conditions. It was therefore important that in each experiment, comparisons were performed between preparations that were similar in all possible parameters, except genotype. We therefore strictly adhered to two principles: first, all experiments were performed in pairwise fashion (Nlgn-1 KO and WT) on the same day or at most, on consecutive days. Second, pairwise experiments were always performed in preparations from homozygous KO and WT littermates. In this manner, effects of uncontrolled variations in experimental conditions were minimized. One last point relates to the genetic perturbation used here. Several recent studies have used RNA interference to suppress Nlgn-1 expression, raising concerns that some of findings reported might be partially related to off-target effects of this methodology [55]. In the current study, the Nlgn-1 expression was eliminated genetically, and consequently, this particular problem did not exist. We cannot rule out the possibility, however, that our findings were partially affected by changes in expression patterns of other (synaptic) molecules that occurred in response to this genetic perturbation.

Much has been learned on synaptic biology and physiology from the genetic elimination of particular synaptic molecules. In many cases, however, the genetic elimination of such molecules was not associated with major changes in synaptic properties as assessed by “single snapshot” techniques, such as immunohistochemistry, electron microscopy, and short-term electrophysiological measurements (for example [13], [64]). In contrast, the methodology used here for examining the constancy of synaptic molecule contents over longer time scales allowed us to expose subtle, but possibly important effects (see below) of such genetic perturbations. This might be taken to suggest that this approach might prove useful for studying how other genetic perturbations affect synaptic physiology over relatively long time scales, and perhaps help to explain phenotypical effects observed at higher levels.

### Nlgn-1 and synaptic tenacity

Numerous studies based on live imaging techniques have revealed that synapses are sites of intense molecular dynamics and that synaptic molecules – receptors, scaffolding, cytoskeletal and signaling molecules alike - as well as synaptic vesicles, patches of active zones, and mitochondria - continuously move in and out of synapses, exhibiting residency times of minutes to hours, and are often interchanged among nearby synapses (reviewed in [65]–[68]). In light of these findings, it might be more appropriate to think of synapses not so much as structures, but as dynamic assemblies at steady states whose constituents are continually exchanged with extrasynaptic pools and shared among nearby synapses. If synapses are so dynamic, as these findings suggest, the capacity of synapses to maintain their individual characteristics, such as size, composition and functional properties, over behaviorally relevant time scales is not obvious. Moreover, if synaptic plasticity is so fundamentally important as widely believed, then synaptic tenacity - the capacity to retain synaptic characteristics at all other times – [39], [44], [69] would seem to be equally important for preserving physiologically relevant modifications and minimizing spurious changes in synaptic function. Within this context, our findings seem to suggest that the loss of Nlgn-1 might have a negative impact on the tenacity exhibited by excitatory synapses: normalized range of change values were greater for PSD-95:EGFP, EGFP:SV2A and SEpH:GluA2 (Figs. 3,5,6); similarly SI decay rates were greater for PSD-95:EGFP and SEpH:GluA2 (Figs. 3,5,6). Moreover, the loss of Nlgn-1 adversely impacted synaptic persistence (Fig. 4). Interestingly, at least where SEpH:GluA2 was concerned, the apparently reduced tenacity exhibited by synapses in networks from Nlgn-1 KO mice depended on the presence of network activity. While it could be argued that this reflects enhanced plasticity in these synapses, this does not agree with prior findings suggesting that well characterized forms of synaptic plasticity (i.e. long-term potentiation) are impaired in these KO mice [36], [70]. We are thus more inclined to think that this reflects an impaired capacity to compensate for use-dependent molecular dynamics (such as enhanced extrasynaptic GluA2 mobility; see [71]) associated with enhanced levels of synaptic transmission. Interestingly, older studies have suggested that synaptic activation is associated with reduced GluA2 mobility at synapses ([72]; see also [73]). Perhaps, this confinement mechanism is compromised in Nlgn-1 KO mice.

In summary, the experiments described here indicate that the loss of Nlgn-1 is associated with a subtle reduction in synaptic tenacity. How this might affect brain function over behavioral time scales is unknown, but it is interesting to note that Nlgn-1 KO mice exhibit deficits in a spatial memory task, in particular 24 hours after learning this task [36]. As intriguing as such ties may seem, however, it should be stressed, given the scale jump between synapses and behavior, that relationships between synaptic tenacity deficits and behavioral impairments, remain tenuous, at best.

## Materials and Methods

### Ethics

All experiments were performed in primary cultures of mouse neurons prepared according to a protocol approved by the “Technion, Israel Institute of Technology Committee for the Supervision of Animal Experiments” (ethics approval number IL-112-09-2009)

### Animals and cell culture

Mice heterozygous to Nlgn-1 deletion (Nlgn-1 KO mice; [13]) were provided as a generous gift by Nils Brose, Max Planck Institute for experimental Medicine, Goettingen, Germany. Cortical neuronal cell cultures were prepared from postnatal day one mice pups as described elsewhere [74]. Briefly, the brains were removed, the entire cortex was separated and chopped, and neurons were isolated by enzymatic digestion and mechanical separation. The dissociated neurons were plated within 8 mm diameter glass cloning cylinders adhered to the center of 22 mm glass coverslips coated with Poly-D-Lysine. Neurons were used for experiments 13–25 days after plating. All preparations were made in parallel from homozygous KO and WT littermate pups, genotyped on day of birth as described below. If a litter did not include both homozygous KO and WT animals, it was not used.

### Genotyping

Genotyping of neonatal mice pups was performed by PCR to identify homozygous KO and WT pups. Primers (Sigma) used for PCR genotyping were as follows: WT: forward GCCCATACGAGAACACAGGT; reverse AGAAAACCCGGCAAGAAAAT (220 base-pair fragment). Nlgn-1 KO: forward GAGCGCGCGCGGCGGAGTTGTTGAC, reverse GTGAGCTGAATCTTATGGTTAGATGGG (415 base-pair fragment). Genotyping was confirmed by preparing western blots of whole-brain tissue from Nlgn-1 KO and WT mice and probing them with an anti Nlgn-1 antibody (Synaptic Systems, Cat. No. 129 013).

### DNA constructs, virus production and transduction

A set of pre and postsynaptic proteins, tagged with fluorescent proteins were introduced into neurons by means of a third-generation lentiviral expression system based on an FUGW backbone [38].These included: PSD-95 tagged with EGFP (PSD-95:EGFP; described in [39]); SV2A tagged with EGFP (EGFP:SV2A; described in [44], [54]); and GluA2 tagged with superecliptic pHluorin (SEpH:GluA2); The latter was constructed by introducing two point mutations in FUGW (to move an XhoI site after the WPRE segment to a location before this element) and then replacing the segment coding for EGFP with a segment coding for Cuticle Protein 3 signal peptide [75], [76], followed by Superecliptic pHluorin (GenBank AY533296.1) and finally rat GluA2 (a generous gift of Yael Stern-Bach, Hebrew University of Jerusalem; GenBank: X54655.1) lacking the first 20 amino acid endogenous signal peptide. This segment, made by de-novo synthesis and sub cloning (Genscript, NJ, USA) was then inserted into modified FUGW using AgeI and XhoI sites. The sequence of all fusion proteins used here was verified by sequencing (Genscript).

Lentiviral particles were produced by transfecting HEK293Tcells with a mixture of three packaging plasmids - pLP1, pLP2, pLP\VSVG (packaging vector mix of the ViraPower four plasmid lentiviral expression system, Invitrogen) and one of the aforementioned expression vectors. Transfection was performed using Lipofectamine 2000 (Invitrogen) in 10 cm plates when the cells had reached 80% confluence. The supernatant (7–8 ml per 10 cm dish) was collected after 48 hr, centrifuged for 15 min at 1550 RCF at 4°C to remove cell debris, filtered through 0.45-µm filters, aliquoted, and stored at −80°C. Transduction of cortical neurons in cell culture was performed on day 5 *in vitro* by adding 3–5µl of the filtered supernatant to each cloning cylinder.

In SEpH:GluA2 experiments in which a minority of Nlgn-1 KO neurons were plated with a majority of neurons prepared in parallel from WT littermates, Nlgn-1 KO mice neurons were exposed to lentiviral particles encoding for SEpH:GluA2 while still in suspension for 2 hours in a humidified CO_2_ incubator, rinsed several times with cell culture media and added at a ratio of 1∶6 (KO∶WT) to cloning cylinders containing neurons from WT littermates plated a few hours earlier.

### Pharmacological manipulations

Reagents were procured from the following sources: CNQX (6-cyano-7 nitroquinoxaline-2,3-dione) from Tocris Bioscience; AP-5 (2-amino-5-phosphonopentanoic acid) from Sigma-Aldrich; TTX (tetrodotoxin) from Alomone Labs. A mixture of CNQX, AP-5, and TTX were diluted in 100 µl medium drawn from the cloning cylinder in which the neurons were grown. Then, the mixture was returned to the cloning cylinder and mixed gently. Final concentrations of these reagents were CNQX- 10 µM, AP-5–50 µM and TTX- 1 µM.

### Long-term imaging

Fluorescence and differential interference contrast images were acquired using a custom-built confocal laser scanning microscope controlled by software written by NEZ, using a 40×, 1.3NA Fluar objective. Time-lapse recordings were performed by averaging 6 frames at 6–12 focal planes spaced 0.8 µm apart. All data were collected at a resolution of 640×480 pixels, at 12 bits/pixel. Data were collected sequentially from up to 6 predefined sites (fields of view), using a robotic XYZ stage to cycle automatically through these sites. Focal drift was corrected before collecting each image stack using the microscope systems “autofocus” feature. Appropriate environmental conditions were provided by heating the chamber base and objective to 36°C and, 35°C respectively, using resistive elements, separate temperature sensors, and controllers, resulting in temperatures of approximately 35°C in the culture medium. The preparations were covered with a custom built enclosure into which a sterile 5% CO_2_, 95% air mixture was streamed. The fluorescent proteins EGFP, and SEpH were excited by using the 488 nm line of an argon laser (JDS Uniphase). Emissions were split between two photomultipliers using a 550 dichroic mirror and filtered at 500–550 nm (EGFP) and 570–610 nm (non-specific fluorescence). Spectral unmixing was then used to remove the contributions of nonspecific fluorescence sources to EGFP/SEpH data.

### Fluorescence recovery after photobleaching

After the collection of baseline images, photobleaching was performed by scanning six preselected 12×12 pixel regions of interest sequentially and repeatedly at 488 nm at high illumination intensity using the confocal microscope systems' acousto-optic tunable filter (AOTF). The photobleaching procedure was performed programmatically using Visual Basic for Applications from within Microsoft Excel. After the photobleaching procedure, fluorescence recovery was recorded for 3 hours by automated time-lapse as described above.

### Data Analysis

Custom software (OpenView) written by one of us (NEZ) was used to track individual synaptic puncta and measure fluorescent intensities of these over time. All analysis was performed on maximal intensity projections of Z-section stacks. Intensities of fluorescent puncta were measured as mean pixel values in 7×7 pixel (∼1×1 µm) rectangular regions centered on individual puncta. These were recentered on the same puncta in the entire time series using automatic tracking algorithms implemented in OpenView (illustrated in Video S1). All tracking was verified and, if necessary, manually corrected. Data collected in this fashion was analyzed using Matlab and Microsoft Excel.

All data collected in FRAP experiments were normalized and corrected for ongoing photobleaching according to the following equation [41]:


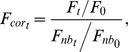


where *F_t_* is the fluorescence at time *t*, *F*_0_ is the pre-photobleaching fluorescence, *F*_nb*t*_ is the average fluorescence intensity of 10 nonbleached puncta at time *t*, and *F_nb_*_0_ is the average fluorescence intensity of the same nonbleached puncta at time *t = 0*. Fluorescence recovery data was then fit to a weighted sum of two exponentials as described in Results.

Long term changes in synaptic contents of fluorescently tagged proteins were quantified using the normalized range of fluorescence changes (“range over mean”) and the Similarity Index (SI) described in Results. To reliably compare SI values from different neurons, we normalized SI values calculated for each neuron to the minimum SI value attainable for that particular neuron. This normalization process was performed according to the following equation:


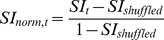


where *SI_t_* is the SI value calculated at time *t*, and *SI_shuffled_* is a value that represents the minimum attainable SI value in an imaginary situation in which the fluorescence of each particular synapse changed to that of some other synapse selected randomly from the entire synaptic population of that neuron. *SI_shuffled_* was calculated by cyclically permutating the last time point measurement vector of puncta fluorescence values and calculating the SI of the shuffled data against the unshuffled *t = 0* fluorescence value vector. This was repeated 10 times for each site and *SI_shuffled_* was taken as the average of these 10 runs. Statistical testing of differences between SI decay curves were calculated as follows: the last 3 SI values of each curve of each neuron were averaged, these average values were pooled for WT and Nlgn-1 KO mice, and compared using a Kolmogorov-Smirnov test. P values greater than 0.05 were considered to be statistically insignificant.

## Supporting Information

Video S1 **Time lapse video a neuron from a Nlgn-1 KO mouse expressing PSD-95:EGFP (23 days in vitro), showing changes over time in the fluorescence intensities of individual PSD-95:EGFP puncta.** Images were collected at 10 minute intervals (112 images, ∼18 hours altogether). Each frame is a maximal intensity projection of 6 sections taken 0.8 µm apart (images for this video were smoothed with a 3×3 low pass filter). Four tracked synapses are enclosed in color coded squares (same as those shown in Figure 3A,B).
(MOV)
